# Inhibitory Activity of Flavonoids against Class I Phosphatidylinositol 3-Kinase Isoforms

**DOI:** 10.3390/molecules16065159

**Published:** 2011-06-21

**Authors:** Dexin Kong, Yanwen Zhang, Takao Yamori, Hongquan Duan, Meihua Jin

**Affiliations:** 1 School of Pharmaceutical Sciences and Research Center of Basic Medical Sciences, Tianjin Medical University, Tianjin 300070, China; 2 Division of Molecular Pharmacology, Cancer Chemotherapy Center, Japanese Foundation for Cancer Research, 3-10-6, Ariake, Koto-ku, Tokyo 135-8550, Japan

**Keywords:** phosphatidylinositol 3-kinase, flavonoid, structure-activity relationship

## Abstract

Class I PI3 Kinase (PI3K) phosphorylates phosphatidylinositol 4,5-bisphophate (PIP2) to generate the second messenger phosphatidylinositol 3,4,5-trisphosphate (PIP3) and therefore plays an important role in fundamental cellular responses such as proliferation. There are four isoforms of class I PI3K which are known to have different functions and relate to various diseases such as cancer and inflammation. Flavonoids are abundant in fruits, vegetables and plant-derived beverages such as tea. So far, various pharmacological effects of flavonoids have been reported. We previously reported that the flavonoid baicalein exhibits potent PI3K-inhibitory activity. Recently we examined the inhibitory activity of eighteen flavonoids against PI3Kα by using an *in vitro* homogenous time resolved fluorescence (HTRF) kinase assay, and deduced their structure-activity relationships by comparing the activities of the analogues. Our result suggests that the number of hydroxyl groups in the A and B rings might promote the activity, while loss of C2-C3 double bond might reduce the activity. Furthermore, the activity against 4 class I PI3K isoforms of some selected flavonoids was investigated, and the results indicate that the flavonoids seem to exhibit more potent activity on PI3Kα and δ isoforms compared with that on PI3Kβ and γ isoforms.

## 1. Introduction

Phosphatidylinositol 3-kinases (PI3Ks) are a family of three classes of lipid kinases that phosphorylate the 3-hydroxyl group of the inositol ring of phosphoinositides [1]. Class I PI3K mainly phosphorylates phosphatidylinositol 4,5-bisphosphate (PIP2) to generate phosphatidylinositol 3,4,5-trisphosphate (PIP3), which recruits and phosphorylates Akt and therefore plays an important role in various cellular responses such as proliferation [2]. This class of PI3K is generally referred to as PI3K because it is much more frequently reported than the other two classes. There are four isoforms of class I PI3K, α, β, δ, and γ. PI3Kα is closely involved in tumorigenesis since a high frequency of mutations in, and amplifications of the PIK3CA gene which encodes p110α, has been found in human cancers [3]. PI3Kβ plays an important role in tumorigenesis of PTEN (phosphatase and tension homolog deleted on chromosome ten) negative cancers [4], besides its another key role in thrombotic diseases [5]. Both PI3Kδ and PI3Kγ are known to be involved in the immune system and various inflammatory responses [2]. 

Development of PI3K inhibitors for cancer therapy has even become a race among pharmaceutical companies. Over ten novel PI3K inhibitors including NVP-BEZ235 (Novartis) and GDC-0941 (Genetech), have demonstrated promising antitumor efficacy on various tumor types [6,7], and are under evaluation in phase I clinical trials [8]. We previously reported the antitumor efficacy *in vitro* and *in vivo* as well as the biochemical inhibition profiles of ZSTK474 [9,10,11,12,13], which has just entered the phase I clinical trials [8]. 

Flavonoids are polyphenolic compounds which are rich in fruit, vegetable, and beverage particularly tea [14]. Accumulated evidences showed they possess various pharmacological activities such as anti-tumor [15], anti-thrombotic [16], and anti-inflammatory effects [17]. Inhibition of 4 PI3K isoforms could lead to such effects. We previously reported that a flavonoid baicalein is a potent PI3K inhibitor [18]. We then predict that other flavonoids may also have PI3K-inhibitory activity. Recently, we examined the PI3K-inhibitory activities of 18 flavonoids which are commercially available. The structure-activity relationships are summarized by comparing their activities.

## 2. Results and Discussion

### 2.1. Inhibitory Activity of the Flavonoids against PI3Kα and the Structure-Activity Relationship

**Figure 1:**
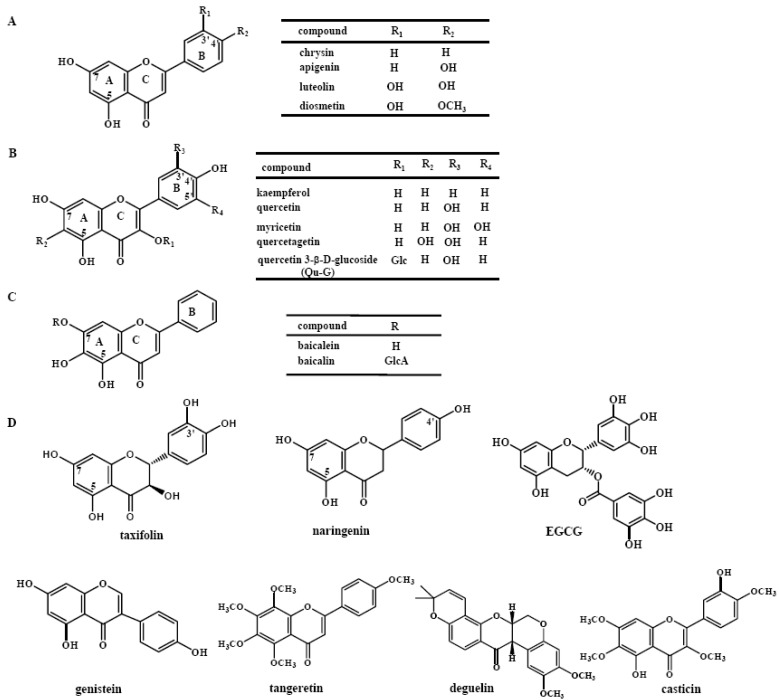
Chemical structures of the flavonoids investigated. (**A**). Chemical structures of chrysin, apigenin, luteolin and diosmetin; (**B**). Chemical structures of kaempferol, quercetin, myricetin, quercetagetin, and quercetin 3-β-D-glucoside; (**C**). Chemical structures of baicalein and baicalin; (**D**). Chemical structures of taxifolin, naringenin, EGCG, genistein, tangeretin, deguelin, and casticin. Glc: glucose; GlcA: glucuronic acid.

**Figure 2:**
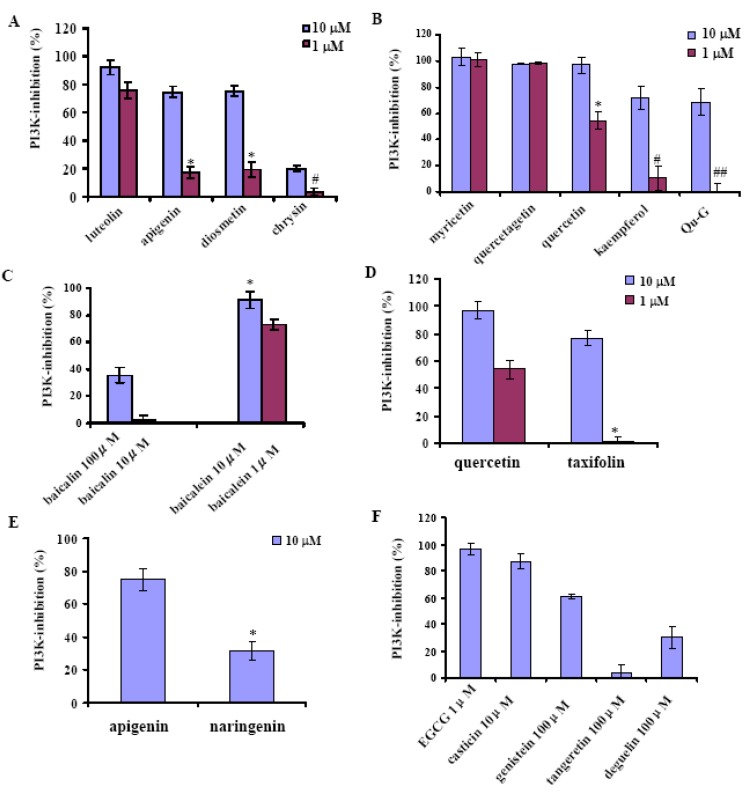
Inhibitory activity of the flavonoids against PI3Kα. Activity is shown as the percentage of PI3Kα activity inhibited. (**A**). Activity of luteolin, apigenin, diosmetin and chrysin at 10 μM and 1 μM, *: p < 0.01 for comparison with luteolin at 1 μM; #: p < 0.01 for comparison with apigenin at 1 μM; (**B**). Activity of myricetin, quercetagetin, quercetin, kaempferol and quercetin 3-β-D glucoside (Qu-G) at 10 μM and 1 μM, *: p < 0.01 for comparison with myricetin at 1 μM; #: p < 0.01 for comparison with quercetin at 1 μM. ##: p < 0.01 for comparison with quercetin at 1 μM; (**C**). Activity of baicalein and baiclin at the respective concentrations, *: p < 0.01 for comparison with baicalin at 10 μM; (**D**). Activity of quercetin and taxifolin at 10 μM and 1 μM; *: p < 0.01 for comparison with quercetin at 1 μM; (**E**). Activity of apigenin and naringenin at 10 μM; (**F**) Activity of EGCG, casticin, genistein, tangeretin and deguelin. *: p < 0.01 for comparison with apigenin. Data are mean ± SD (n = 3), representative of 3 independent experiments.

The structures of the investigated flavonoids are shown in Figure 1. They include flavones (chrysin, apigenin, luteolin, diosmetin, baicalein, baicalin, and tangeretin), flavonols (kaempferol, quercetin, myricetin, quercetagetin), a flavanone (naringenin), an isoflavone (genistein), a flavanonol (taxifolin) and other analogues (quercetin-3-β-D-glucoside, EGCG, deguelin, casticin). Their activities on the recombinant PI3Kα are indicated in Figure 2. Figure 2A show s the inhibitory activities of four flavones. Among the four flavones, 1 μM of luteolin exhibits most potent activity with 75.8% PI3Kα inhibited, in contrast to chrysin which exhibits far less potency at the same concentration. Correlation of the activities with the chemical structures shows that the activity is promoted as the number of the hydroxyl group in B ring increases, because luteolin shows higher activity than both apigenin and diosmetin (p < 0.01), while chrysin indicates less activity than apigenin (p < 0.01) (Figure 1A and Figure 2A

The inhibitory activities of some flavonols such as kaempferol, quercetin, myricetin, quercetagetin, and a glucoside of quercetin named quercetin 3-β-D-glucoside (Qu-G) at 10 and 1 μM are shown in Figure 2B. Both myricetin and quercetagetin almost completely inhibit the activity of PI3Kα at 1 μM, more potent than quercetin (p < 0.01 for both) which inhibits 54.1% of PI3Kα at 1 μM. In contrast, kaempferol indicates far weaker activity than quercetin (p < 0.01) for PI3Kα inhibition. The results indicate that the number of hydroxyl groups in both the A and B rings has a positive correlation with the PI3Kα inhibitory activity. In contrast, the existence of hydroxyl groups in C ring might not increase the activity, since luteolin and apigenin do not exhibit lower activity than quercetin and kaempferol, respectively. (Figure 1A, 1B, Figure 2A and 2B). On the other hand, glycosidation at C-3 might decrease the PI3K inhibitory potency, since the activity of Qu-G is much weaker than that of quercetin (p < 0.01) (Figure 2B). Glucuronidation seems to highly reduce the activity since baicalein inhibits 91.5% PI3Kα, while its glucuronide baicalin only inhibits 35.5% at 10 μM (Figure 1C and Figure 2C

The activities of flavone tangeretin and other types of flavonoids like taxifolin (flavanonol), naringenin (flavanone), genistein (isoflavone), EGCG, deguelin and casticin were also investigated. Comparison of the activities of quercetin and taxifolin (p < 0.01, Figure 1A, 1D and Figure 2D), apigenin and naringenin (p < 0.01, Figure 1A, 1D and Figure 2E) suggests the loss of the C2-C3 double bond might decrease the inhibitory potency. As shown in Figure 2F, EGCG exhibits potent PI3K inhibitory activity at 1 μM, and casticin indicates activity at 10 μM. In contrast, genistein and deguelin only show weak PI3K inhibition at 100 μM. Tangeretin (Figure 1D) does not show inhibitory activity even at 100 μM (Figure 2F, suggesting the hydroxyl group might be a must for the PI3Kα inhibitory activity of flavonoids. As mentioned above, our study has provided information about the structure-activity relationship of the flavonoids, which is important for design of more potent derivatives as PI3K inhibitors.

### 2.2. Selectivity of the Flavonoids in Inhibition against Four Class I PI3K Isoforms

**Figure 3:**
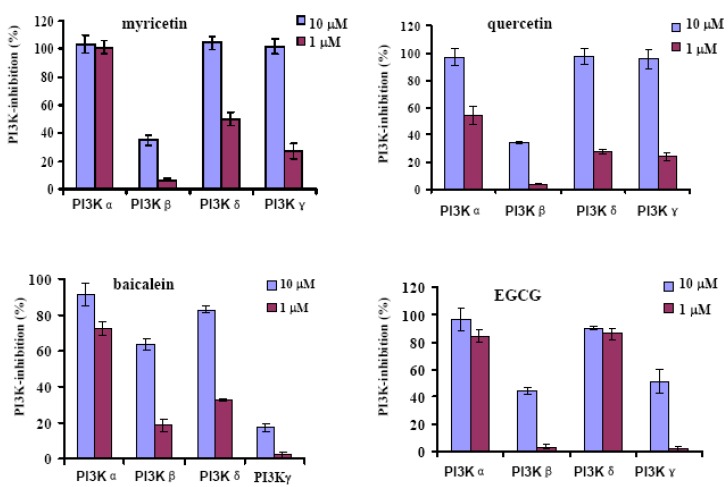
Inhibitory activity of the selected flavonoids against each class I PI3K isoform.Activity of the compounds at 10 μM and 1 μM is shown respectively as the percentage of PI3K activity inhibited (PI3K-inhibition). Data are mean ± SD (n = 3), representative of 3 independent experiments.

Four class I PI3K isoforms have been reported to possess their respective functions [3,19,20]. To investigate the selectivity of the flavonoids for the four isoforms, some flavonoids such as myricetin, quercetin, baicalein and EGCG were assayed for their inhibition against other three PI3K isoforms. As shown in Figure 3, the compounds seem to inhibit PI3Kα and δ more potently than PI3Kβ and γ. Since PI3Kβ is known to play a key role in PTEN negative cancers [4], these flavonoids might be more applicable for treatment of patients with PTEN positive cancers.

We noted that EGCG has just been reported as a dual PI3K/mTOR inhibitor [21], supporting our result about its PI3K-inhibitory activity. We previously reported the PI3K-inhibitory activity of baicalein [18], this time we also investigated the activity of its glucuronide -baicalin and found that baicalin possesses far weaker activity than baicalein. Since orally administered baicalein would be transformed to baicalin after metabolism in the body [22], increased dose might be needed for baicalein to target PI3K. The inhibition mode of some flavonoids has been reported [21]. Biochemical analysis showed that they should be ATP-competitive PI3K inhibitors, and the molecular docking study suggested they bind with ATP-binding pocket of PI3K [21].

Antitumor, anti-thrombotic and anti-inflammatory activities of the flavonoids have been frequently reported [15,16,17]. Our study indicates that the flavonoids inhibit four class I PI3K isoforms, which are known to be involved in diseases such as tumor, thrombosis and inflammation, suggesting that PI3K might be one molecular target of the flavonoids when used for treatment of such diseases. 

Flavonoids are generally much less toxic compared with current antitumor drugs in the clinic. The consumption of total flavonoids may attain as high as 100 mg/day [23]. Plasma concentration of some flavonoids could be over 5 μM after a single ingestion of orange juice or 300 mL of green tea [24]. Therefore, such flavonoids are expected to be used for cancer prevention.

## 3. Experimental

### 3.1. General

Eighteen flavonoids were examined for the inhibitory activity against PI3Kα, by using a novel assay method- homogenous time resolved fluorescence (HTRF) kinase assay. By comparison of the activities of the derivatives with each other, the structure-activity relationship was summarized. Some compounds were selected to examine the activity against 4 class I PI3K isoforms by using the recombinant PI3Ks and HTRF assay method. 

### 3.2. Materials

Luteolin, chrysin, (+)-taxifolin and (±)-naringenin were purchased from Alexis; Genistein, (-)-epigallocatechin gallate, (-)-deguelin, adenosine 5’-triphosphate (ATP) disodium salt and DL-dithiothreitol (DTT) were from Sigma; quercetagetin and diosmetin were from Chromadex Inc.; apigenin, baicalein, baicalin and tangeretin were from WAKO; myricetin, kaempferol and quercetin-3-β-D-glucoside were from Fluka; casticin was from Indofine Chemical. The PI3K Homogenous Time Resolved Fluorescence (HTRF) Assay Kit and human recombinant PI3Kα, β, δ and γ were purchased from Millipore (Billerica, MA).

### 3.3. Homogenous Time Resolved Fluorescence (HTRF) Assay for Determination of PI3K Activity

To determine the PI3K activity with or without the presence of the flavonoids, the HTRF assay was carried out as described previously with a small modification [9]. Briefly, various concentrations of the flavonoids were incubated with the recombinant PI3Kα, PI3Kβ, PI3Kδ and PI3Kγ in the assay buffer supplemented with 10 μM (final concentration) of PIP2 in the wells of a 384-well plate at room temperature. Reaction was initiated by addition of 10 μM ATP and was stopped after 30 min of incubation by adding the stop solution containing EDTA and biotin-PIP3. Detection buffer was then added and the resulting mixture was further incubated for 14 h. Signals from the wells were read using the EnVision 2103 Multilabel Reader (PerkinElmer, Wellesley, MA). The PI3K inhibitory activity of each compound was calculated according to the following formula: PI3K-inhibition (%) = (plus-enzyme control – sample) / (plus-enzyme control – minus-enzyme control) × 100. For the plus-enzyme control, the kinase was incubated with PIP2 and ATP in the absence of flavonoid, and for the minus-enzyme control, PIP2 was incubated with ATP in the absence of kinase and flavonoid. Representative data from three independent experiments, each carried out in triplicate, were used for plotting the graph. 

## 4. Conclusions

We examined the inhibitory activity of eighteen flavonoids against PI3K using an HTRF kinase assay. The flavonoids exhibited PI3K inhibition with different potency. Some structure-activity relationship was then obtained. Furthermore, the flavonoids seem to exhibit more potent activity on PI3Kα and δ isoforms compared with that on PI3Kβ and γ isoforms.

## References

[1] Toker A., Cantley L.C. (1997). Signalling through the lipid products of phosphoinositide-3-OH kinase. Nature.

[2] Kong D., Yamori T. (2008). Phosphatidylinositol 3-kinase inhibitors: promising drug candidates for cancer therapy. Cancer Sci..

[3] Samuels Y., Wang Z., Bardelli A., Silliman N., Ptak J., Szabo S., Yan H., Gazdar A., Powell S.M., Riggins G.J. (2004). High frequency of mutations of the PIK3CA gene in human cancers. Science.

[4] Wee S., Wiederschain D., Maira S.M., Loo A., Miller C., de Beaumont R., Stegmeier F., Yao Y.M., Lengauer C. (2008). PTEN-deficient cancers depend on PIK3CB. Proc. Natl. Acad. Sci. USA.

[5] Jackson S.P., Schoenwaelder S.M., Goncalves I., Nesbitt W.S., Yap C.L., Wright C.E., Kenche V., Anderson K.E., Dopheide S.M., Yuan Y. (2005). PI 3-kinase p110beta: A new target for antithrombotic therapy. Nat. Med..

[6] Maira S.M., Stauffer F., Brueggen J., Furet P., Schnell C., Fritsch C., Brachmann S., Chene P., De Pover A., Schoemaker K. (2008). Identification and characterization of NVP-BEZ235, a new orally available dual phosphatidylinositol 3-kinase/mammalian target of rapamycin inhibitor with potent *in vivo* antitumor activity. Mol. Cancer Ther..

[7] Folkes A.J., Ahmadi K., Alderton W.K., Alix S., Baker S.J., Box G., Chuckowree I.S., Clarke P.A., Depledge P., Eccles S.A. (2008). The identification of 2-(1H-indazol-4-yl)-6-(4-methanesulfonyl-piperazin-1-ylmethyl)-4-morpholin-4-yl-thieno[3,2-d]pyrimidine (GDC-0941) as a potent, selective, orally bioavailable inhibitor of class I PI3 kinase for the treatment of cancer. J. Med. Chem..

[8] Kong D., Yamori T. (2009). Advances in development of phosphatidylinositol 3-kinase inhibitors. Curr. Med. Chem..

[9] Kong D., Yamori T. (2007). ZSTK474 is an ATP-competitive inhibitor of class I phosphatidylinositol 3 kinase isoforms. Cancer Sci..

[10] Kong D., Okamura M., Yoshimi H., Yamori T. (2009). Antiangiogenic effect of ZSTK474, a novel phosphatidylinositol 3-kinase inhibitor. Eur. J. Cancer.

[11] Kong D., Yaguchi S., Yamori T. (2009). Effect of ZSTK474, a novel phosphatidylinositol 3-kinase inhibitor, on DNA-dependent protein kinase. Biol. Pharm. Bull..

[12] Yaguchi S., Fukui Y., Koshimizu I., Yoshimi H., Matsuno T., Gouda H., Hirono S., Yamazaki K., Yamori T. (2006). Antitumor activity of ZSTK474, a new phosphatidylinositol 3-kinase inhibitor. J. Natl. Cancer Inst..

[13] Kong D., Dan S., Yamazaki K., Yamori T. (2010). Inhibition profiles of phosphatidylinositol 3-kinase inhibitors against PI3K superfamily and human cancer cell line panel JFCR39. Eur. J. Cancer.

[14] Crozier A., Burns J., Aziz A.A., Stewart A.J., Rabiasz H.S., Jenkins G.I., Edwards C.A., Lean M.E. (2000). Antioxidant flavonols from fruits, vegetables and beverages: measurements and bioavailability. Biol. Res..

[15] Kanadaswami C., Lee L.T., Lee P.P., Hwang J.J., Ke F.C., Huang Y.T., Lee M.T. (2005). The antitumor activities of flavonoids. In Vivo.

[16] Peluso M.R. (2006). Flavonoids attenuate cardiovascular disease, inhibit phosphodiesterase, and modulate lipid homeostasis in adipose tissue and liver. Exp. Biol. Med. (Maywood).

[17] Middleton E. (1998). Effect of plant flavonoids on immune and inflammatory cell function. Adv. Exp. Med. Biol..

[18] Kong D., Yamazaki K., Yamori T. (2010). Discovery of phosphatidylinositol 3-kinase inhibitory compounds from the Screening Committee of Anticancer Drugs (SCADS) library. Biol. Pharm. Bull..

[19] Rommel C., Camps M., Ji H. (2007). PI3K delta and PI3K gamma: partners in crime in inflammation in rheumatoid arthritis and beyond?. Nat. Rev. Immunol..

[20] Jia S., Liu Z., Zhang S., Liu P., Zhang L., Lee S.H., Zhang J., Signoretti S., Loda M., Roberts T.M., Zhao J.J. (2008). Essential roles of PI(3)K-p110beta in cell growth, metabolism and tumorigenesis. Nature.

[21] Van Aller G.S., Carson J.D., Tang W., Peng H., Zhao L., Copeland R.A., Tummino P.J., Luo L. (2011). Epigallocatechin gallate (EGCG), a major component of green tea, is a dual phosphoinositide-3-kinase/mTOR inhibitor. Biochem. Biophys. Res. Commun..

[22] Akao T., Kawabata K., Yanagisawa E., Ishihara K., Mizuhara Y., Wakui Y., Sakashita Y., Kobashi K. (2000). Baicalin, the predominant flavone glucuronide of scutellariae radix, is absorbed from the rat gastrointestinal tract as the aglycone and restored to its original form. J. Pharm. Pharmacol..

[23] Hertog M.G., Kromhout D., Aravanis C., Blackburn H., Buzina R., Fidanza F., Giampaoli S., Jansen A., Menotti A., Nedeljkovic S. (1995). Flavonoid intake and long-term risk of coronary heart disease and cancer in the seven countries study. Arch. Intern. Med..

[24] Erlund I., Meririnne E., Alfthan G., Aro A. (2001). Plasma kinetics and urinary excretion of the flavanones naringenin and hesperetin in humans after ingestion of orange juice and grapefruit juice. J. Nutr..

